# Legal precarity as culturally situated acculturative stress: social support and psychological security among Akha-Hani cross-border wives in Southwest China

**DOI:** 10.3389/fpsyg.2026.1926621

**Published:** 2026-09-30

**Authors:** Shu Li, Eshah A. Wahab

**Affiliations:** Department of Anthropology and Sociology, Faculty of Arts and Social Sciences, Universiti Malaya, Kuala Lumpur, Malaysia

**Keywords:** acculturative stress, Akha-Hani, cross-border marriage, cultural psychology, legal precarity, psychological security, social support

## Abstract

This article examines how legal precarity becomes acculturative stress and how relational support shapes psychological security among Akha-Hani cross-border wives in Southwest China. Existing acculturation research has established that migration involves psychological and sociocultural adjustment, but less is known about how stress develops when substantial cultural proximity coexists with legal and administrative uncertainty. Based on prolonged field engagement between 2022 and 2024 and formal ethnographic data collection from July to September 2024 in Xishuangbanna, the study draws on 28 in-depth interviews, four focus group discussions involving 29 additional participants, and participant observation. The findings show that shared Akha-Hani language, kinship, and ritual familiarity reduced some forms of interpersonal and cultural distance, but did not remove stress associated with documentation, public procedures, mobility, and family futures. Legal precarity became psychologically consequential through uncertainty, dependence on others, restricted agency, and reduced predictability in everyday family life. Mandarin was important mainly in public and institutional settings rather than as a general barrier within Akha-Hani households. Emotional, practical, and informational support from husbands, relatives, peers, and local networks helped women manage these pressures and strengthen psychological security. However, support was sometimes ambivalent when immediate assistance also maintained dependence on particular family relationships. The study contributes to cultural and migration psychology by showing how institutional insecurity can generate acculturative stress even under conditions of substantial cultural proximity.

## 1 Introduction

Cross-border marriage is not only a demographic or legal process. For women who move into a new family and village across a national border, marriage can also become a process of cultural and psychological adjustment. It involves learning new routines, negotiating language use, building trust with in-laws, and gaining recognition from local people. Acculturation research has long shown that migration may produce both psychological and sociocultural challenges (Berry, 1997, 2001; Ward and Kennedy, 1999). However, these challenges are not experienced in the same way everywhere. They are shaped by legal status, family structure, gendered responsibilities, language resources, and the wider social and institutional conditions of settlement.

Acculturative stress is increasingly understood as context dependent rather than as a direct consequence of cultural difference. Research has linked acculturative stress with language resources, family relations, social support, discrimination, fear, and wider settlement conditions (Bekteshi and Kang, 2020; Lerias et al., 2025). This perspective is important because migrants who share substantial cultural familiarity with a receiving community may still face psychological strain when other areas of everyday life remain uncertain. Marriage migration adds another layer because adaptation takes place through intimate family relations as well as through public and institutional encounters.

Research on marriage migration in Asia further shows that integration does not follow a single or uniform trajectory across family, social, and institutional settings (Yeung and Mu, 2020; Chang, 2020; Chiu and Yeoh, 2021; Lin and Shih, 2026). Studies of China’s ethnic borderlands have documented unstable legal status and limited formal recognition among some foreign wives (Barabantseva, 2018; Grillot, 2015). Wider migration research on liminal legality, deportability, and precarious migratory status also demonstrates that uncertain legal positions can shape fear, dependence, mobility, and access to social rights (De Genova, 2002; Menjívar, 2006; Goldring et al., 2009). These studies suggest that psychological adaptation should be examined not only through cultural distance, but also through the conditions that shape predictability and agency in everyday life.

The Akha-Hani case is especially useful for examining this problem because cross-border marriages frequently occur under conditions of substantial cultural proximity. Akha-Hani communities extend across Southwest China and neighbouring areas of Myanmar and Laos, and historical and ethnographic research documents important continuities in language, ancestry, ritual practice, and transborder social relations (He, 2012; Zhang, 2013). Regional research also shows that cross-border marriage in Yunnan is often embedded in shared ethnic, linguistic, and cultural worlds that predate contemporary state boundaries (Shen, 2023). Such proximity can reduce some interpersonal and symbolic distance. It does not, however, remove differences in nationality, administrative procedures, public institutions, or the linguistic demands of state-facing encounters.

The Akha-Hani case therefore raises a specific psychological question. Cultural familiarity may reduce some demands of adaptation, while legal and administrative uncertainty can continue to affect predictability, independence, and family planning. This article examines how legal precarity becomes acculturative stress among Akha-Hani cross-border wives, how culturally organised forms of social support help women manage this uncertainty, and under what conditions such support strengthens psychological security or maintains dependence. Local recognition remains relevant because it can reduce social uncertainty, but the principal analytical focus is the relationship among institutional insecurity, acculturative stress, relational support, and psychological security. By examining these processes in a rural ethnic borderland, the study also broadens the range of settings represented in cultural psychology (Henrich et al., 2010).

## 2 Theoretical framework

### 2.1 Legal precarity as culturally situated acculturative stress

Acculturation research provides the main psychological foundation for this study. Berry (1997, 2001) defines acculturation as a process of cultural and psychological change that occurs when individuals or groups come into sustained contact with another cultural setting. This process may involve language learning, emotional strain, identity negotiation, and changes in everyday relationships. Acculturative stress refers to stress reactions that emerge when people face new cultural demands and uncertain forms of belonging. For migrants, this stress is often shaped by language barriers, unfamiliar social rules, social distance, and access to support.

Ward and Kennedy (1999) distinguish between psychological adaptation and sociocultural adaptation. Psychological adaptation refers to emotional wellbeing and a sense of security. Sociocultural adaptation refers to the ability to manage everyday tasks and social interaction in the host setting. This distinction is important for the present study because cross-border wives may learn local rituals and household routines, but this learning does not automatically remove anxiety or insecurity. In village life, psychological adaptation and sociocultural adaptation often develop together. A woman may feel safer when she can speak the local dialect, follow ritual procedures, or receive help from her husband’s kin.

This distinction also points to the need for a multidimensional view of adaptation. Research on Asian marriage migration shows that incorporation can vary across social, family, and institutional settings rather than following one uniform trajectory (Yeung and Mu, 2020). Research with marriage-based immigrant women similarly treats acculturation as involving multiple domains, including language, social interaction, cultural heritage, and everyday practice (Chen et al., 2021). Cultural familiarity within the household or ethnic community therefore cannot be assumed to provide equivalent competence or security in public and institutional settings.

Recent migration research further supports a contextual understanding of psychological stress and adaptation (Ozola-Cīrule and Martinsone, 2025). Psychological strain has been associated with fear, distress, emotion regulation, meaning-making, and depression across different migrant populations (Starck et al., 2020; López-de-León et al., 2025; Soufi Amlashi and Forstmeier, 2025). Studies of adaptation and social support also show that perceived support can buffer acculturative stress, while ethnic identity and social distance shape adaptation in culturally diverse settings (Chen, 2025; Hai et al., 2025). Systematic and scoping reviews similarly indicate that language resources, family relationships, social support, fear, and the wider settlement environment can shape how acculturative stress is experienced (Bekteshi and Kang, 2020; Lerias et al., 2025).

In the present study, legal precarity refers to unstable or incomplete security in state-recognised marital status, identity documentation, residence, mobility, public services, and administrative procedures that affect family life. The concept does not imply that all women occupied the same documentary position. Nor is legal precarity treated as equivalent to acculturative stress. It becomes psychologically relevant when administrative uncertainty reduces the predictability of everyday life or increases dependence on other people.

Several processes connect legal precarity with acculturative stress. Uncertainty over documentation, residence, mobility, child registration, or service access can make future planning less predictable. Complex procedures may also increase reliance on husbands, relatives, interpreters, or local intermediaries. Where women have limited capacity to complete these tasks independently, uncertainty may be accompanied by reduced control, anticipatory fear, and concern about family futures. Legal precarity is therefore treated as an institutional condition that can generate acculturative stress through uncertainty, dependence, and restricted agency.

This article extends this literature by focusing on cross-border wives in rural Akha-Hani communities. In this setting, acculturative stress is not caused by cultural difference alone. It is also shaped by legal precarity, language dependence, household expectations, ritual obligations, and village evaluation. Although some women from Myanmar and Laos share ethnic and linguistic ties with Chinese Akha-Hani communities, shared ethnicity does not remove adaptation pressure. After marriage, women still need to learn local village rules, manage relations with in-laws, participate in ritual events, and deal with state registration procedures. Therefore, acculturative stress is treated here as a culturally situated experience rather than only an individual psychological state.

Language dependence is treated as domain specific rather than as a general marker of cultural distance. Shared Akha-Hani speech can substantially reduce linguistic barriers within households and kin networks. Mandarin becomes more consequential in township markets, schools, healthcare settings, banks, police stations, and other public or state-facing encounters. Limited Mandarin proficiency therefore does not create legal precarity by itself. It may intensify the psychological consequences of administrative uncertainty when women require assistance to understand procedures, communicate with institutions, or act independently.

### 2.2 Relational support as stress buffering

Social support is the second key concept in this study. The stress-buffering model proposes that social support can reduce the psychological impact of stressful conditions by providing emotional, practical, and informational resources (Cohen and Wills, 1985). Research with immigrant populations also links family and social resources with adaptation, community integration, and wellbeing (Hombrados-Mendieta et al., 2019; El Khayat et al., 2024), while studies of acculturative stress identify family and social support as important buffering resources (Bekteshi and Kang, 2020; Chen, 2025). In this study, emotional support includes reassurance and companionship from husbands, relatives, peer women, and natal kin. Practical support includes childcare, transport, shared labour, and assistance with everyday tasks. Informational support includes translation, explanations of procedures, ritual guidance, and advice about local practices.

Support is treated as relational because it is enacted through everyday interaction rather than understood only as an individual perception. It can reduce uncertainty by making unfamiliar or institutionally difficult situations more manageable. However, support is not assumed to be uniformly protective. Reliance on husbands, senior relatives, or intermediaries may reduce immediate stress while maintaining dependence in other areas of life. The protective value of support therefore depends partly on whether assistance increases women’s capacity to act independently.

Research on meaning-making and emotion regulation further suggests that the consequences of acculturative stress cannot be separated from the resources through which people interpret and manage difficult situations (Soufi Amlashi and Forstmeier, 2025). The present study does not measure these psychological processes statistically, but examines how uncertainty becomes more or less manageable through culturally organised relationships.

Social identity, group membership, and post-migration belonging remain relevant to psychological security because they can provide social meaning, support, and a more secure social position (Haslam et al., 2009; Hu and Cheung, 2024; Trotter and Woodcock, 2025). In the present article, however, local recognition is not developed as a separate theory of post-marital belonging. It is treated more narrowly as one relational condition that can reduce social uncertainty and contribute to psychological security. This distinction keeps recognition analytically separate from emotional, practical, and informational support.

### 2.3 Gendered psychological security and constrained agency

Gendered marriage migration provides an important context for understanding psychological security among Akha-Hani cross-border wives. Cross-border marriage connects intimacy, mobility, family responsibility, and power (Constable, 2005). Studies of China’s borderlands further show that foreign wives may experience unstable legal status, incomplete documentation, and dependence on local arrangements (Barabantseva, 2018; Grillot, 2015). Research on Vietnamese marriage migrants in Taiwan and South Korea also demonstrates that gender systems shape integration across private and public spheres through expectations attached to wives and daughters-in-law (Chang, 2020).

The Akha-Hani case requires attention to these gendered positions because women from Myanmar and Laos enter marital households as wives, daughters-in-law, mothers, agricultural workers, and carers. Domestic labour, ritual participation, childcare, respect for elders, language learning, and family reputation shape their everyday responsibilities. These expectations matter psychologically when they increase pressure to perform roles correctly, limit independent decision-making, or make access to support dependent on household relationships.

Legal precarity makes this process more complex. It refers to unstable or incomplete access to formal recognition, identity documents, mobility rights, and public services. In borderland marriages, legal precarity may involve uncertain marriage registration, limited residence status, parentage verification, household registration difficulties, and restricted access to services for spouses or children. These issues are not only legal problems. They may become sources of fear, dependence, and everyday stress.

For Akha-Hani cross-border wives, the border is not only a line between China, Myanmar, and Laos. It is also lived through documents, language, household authority, ritual rules, public service access, and village evaluation. Shared language, ancestry, and ritual background may reduce some cultural distance. However, shared ethnicity does not remove the border. After marriage, women still need to enter Chinese registration systems, local household expectations, village moral rules, and public service arrangements.

The framework of this article has three connected components. First, legal precarity is treated as an institutional condition that can generate acculturative stress through uncertainty, dependence, reduced control, and concern about family futures. Second, social support is treated as a relational buffering process through which emotional, practical, and informational resources make stressful situations more manageable. Third, psychological security is treated as an outcome that increases when women experience greater predictability, reliable support, communicative competence, and room for independent action.

These processes do not form a simple linear sequence. Cultural familiarity can reduce some interpersonal demands while administrative uncertainty remains psychologically consequential. Support can strengthen security, but the same relationship can also maintain dependence. Local recognition may contribute to social confidence, but it is treated here as part of the relational context rather than as a separate theory of belonging.

Social Exchange Theory is used only as a supporting lens (Blau, 1964). It helps explain how access to help, resources, reciprocity, and dependence can shape household relationships. The principal analytical focus remains cultural psychology, particularly the relationship among acculturative stress, relational support, agency, and psychological security.

## 3 Materials and methods

### 3.1 Research design

This article reports an article-specific qualitative analysis from a broader doctoral ethnographic project on Akha-Hani cross-border marriage in Xishuangbanna. The broader project examined cultural adaptation, identity negotiation, mobility, and policy conditions. The present article focuses specifically on how legal and administrative uncertainty, language dependence, gendered household expectations, and relational support shape acculturative stress and psychological security among cross-border wives.

This study adopted an interpretive ethnographic design because the research question concerns how institutional conditions acquire psychological meaning through everyday practices, relationships, and situated interaction. Ethnography is appropriate for examining social meanings and practices through sustained engagement with participants in their everyday settings (Reeves et al., 2008; Hammersley and Atkinson, 2019). In the present article, interviews and focus group discussions provided the principal textual materials, while participant observation was used to contextualise how these meanings were enacted in household, agricultural, ritual, and village settings.

The purpose was not to estimate the prevalence of acculturative stress, but to examine how institutional uncertainty, gendered expectations, language use, and social relationships became psychologically meaningful in everyday family and village life.

The study follows a qualitative interpretive approach. It uses interviews, focus group discussions, and participant observation to understand how cross-border wives describe and experience adaptation. The analysis does not treat culture as a fixed background. Instead, it examines how cultural expectations are enacted through documents, language, household labour, ritual participation, and local recognition.

### 3.2 Research sites and fieldwork context

The study was conducted in Akha-Hani communities in Xishuangbanna, Yunnan Province, Southwest China. Fieldwork focused on three townships: Gelanghe, Damenglong, and Daluo. These sites were selected because they include Akha-Hani villages where cross-border marriages with women from Myanmar and Laos are part of local family life. They also provide suitable settings for observing the relationship between marriage, kinship, language, ritual participation, agricultural labour, and local forms of recognition.

The first author maintained repeated contact with the field between mid-2022 and mid-2024. This earlier engagement provided contextual familiarity and supported field access, but it was not treated as part of the formal analytical corpus. Formal recruitment, interviews, focus group discussions, and concentrated participant observation were conducted from July to September 2024 after ethical approval had been obtained.

The three townships were not treated as strictly comparative cases. Instead, they were used as a multi-sited fieldwork setting. This approach made it possible to observe similar processes across different villages and kinship networks. It also reduced the risk of relying on one village or one family network only.

### 3.3 Participants and sampling

Eligible participants were adults with direct experience of Akha-Hani cross-border marriage or close and sustained knowledge of the family and community contexts in which these marriages were lived. Primary participants included women and men directly involved in cross-border marriages. Contextual participants included adult relatives, elders, traditional headmen, village leaders, and community members with sustained interaction with cross-border families.

Participants were recruited through purposive and snowball sampling. Public recruitment was not used because questions concerning marital registration, documentation, mobility, and family status could be sensitive in small village settings. Initial access relied on several trusted contacts, kinship networks, and participant referrals. These contacts facilitated introductions but did not determine eligibility or final inclusion. Consent was obtained directly by the researcher.

The study involved 57 unique participants across the three field sites. 28 participated in individual interviews, while 29 different participants joined four focus group discussions. No participant was counted in both datasets. The sample was designed for qualitative depth and variation rather than statistical representation. It provided perspectives across different marital, family, generational, and community positions relevant to the research questions.

Sample adequacy was assessed in relation to information power rather than statistical representativeness or a fixed numerical threshold. The study had a focused research aim, participants with direct or sustained knowledge of cross-border marriage, multiple participant positions across three field sites, and substantial interview and focus group material. These features increased the information power of the dataset for addressing the research questions (Malterud et al., 2016). The final sample of 57 participants was therefore considered adequate for the article-specific qualitative analysis while preserving variation across marital, family, generational, and community positions (Table 1).

**Table 1:** Participant categories by field site.

Participant category	Gelanghe township	Damenglong township	Daluo township	Total
Cross-border wives	11	3	2	16
Chinese husbands	10	4	0	14
Divorced participants	3	0	0	3
Adult children of cross-border families	2	0	0	2
Parents or parents-in-law	2	0	0	2
Siblings and extended family members	4	1	1	6
Village leaders, elders, and traditional headmen	6	1	0	7
Community members	2	0	5	7
Total	40	9	8	57

With participants’ consent, interviews and focus group discussions were audio recorded and, where available, audiovisual recordings were retained to assist transcription and the interpretation of contextual or non-verbal information. These recordings were not analysed as a separate dataset.

### 3.4 Data collection

Semi-structured interviews were used to collect individual narratives about marriage, adaptation, stress, support, and belonging. Most interviews lasted between 60 and 120 min. They were conducted in participants’ homes or in other quiet spaces chosen by participants. The interview format allowed participants to describe their experiences in their own terms, while also allowing the researcher to follow up on key themes.

The interview guide covered marriage history, reasons for cross-border marriage, legal status, documentation experience, language use, household roles, relationships with husbands and in-laws, ritual participation, village interaction, family support, kinship ties across the border, and feelings of uncertainty, security, and belonging. The guide was flexible. When participants raised unexpected issues, such as fear of official attention, difficulties in public service access, or family conflict, follow-up questions were asked only when it was safe and appropriate.

Four focus group discussions involved 29 participants. Three groups contained seven participants and one group contained eight. The four focus groups differed in composition. FGD1 included a cross-border spouse together with relatives and other community members; FGD2 focused on female cross-border spouses; FGD3 consisted of men with current or previous experience of cross-border marriage; and FGD4 consisted of local community members, including elders and village leaders. This design provided perspectives from both people directly involved in cross-border marriages and members of the wider community.

Participant observation complemented the interview and focus group data. Repeated visits were made to village households, agricultural work settings, and ritual or festival events. Observation focused on how cross-border wives participated in daily life, used language, interacted with husbands and in-laws, received support, and were evaluated by family or village members. These observations were used to contextualise and support the interpretation of interview and focus group accounts rather than as a separate coded dataset.

### 3.5 Language, interpretation, and transcription

Interviews and focus group discussions were conducted in Standard Mandarin Chinese, hereafter Mandarin, when participants could communicate directly with the researcher. Field engagement indicated that Mandarin was routinely used by younger local residents and was also widely spoken among middle-aged residents. Proficiency was more uneven among a small number of older residents and among foreign spouses from Myanmar and Laos. Some foreign spouses could communicate in Mandarin, whereas others relied mainly on Akha-Hani.

When participants preferred Akha-Hani or did not have sufficient Mandarin for an in-depth discussion, a local bilingual Akha-Hani and Mandarin interpreter provided consecutive interpretation. The interpreter also facilitated some initial introductions where prior local trust was useful for field access. However, the interpreter did not determine participant eligibility, select the final sample, or obtain consent on behalf of the researcher. Consent was obtained directly by the researcher.

With participants’ consent, interviews and focus group discussions were recorded and transcribed in Chinese. Akha-Hani language segments were interpreted into Mandarin and incorporated into the Chinese transcript. Quotations selected for publication were translated from Chinese into English by the first author. Interpreted accounts were treated as mediated qualitative data, and locally specific terms were checked against field context and follow-up clarification when available.

### 3.6 Data analysis

The principal coded materials were interview and focus group transcripts. Participant observation was used contextually to support interpretation and to compare emerging themes with everyday practices observed during fieldwork. Following Braun and Clarke (2006), the analysis moved from repeated reading of the data to initial coding, theme development, review, and interpretation.

The first stage involved open coding. Interview and focus group transcripts were read repeatedly to identify initial codes related to stress, uncertainty, language use, household labour, ritual participation, documentation, support, recognition, and belonging. The second stage involved focused coding. Initial codes were grouped into broader themes, including legal precarity, language dependence, household expectations, family support, village recognition, psychological security, and social belonging. The third stage involved theoretical interpretation. The focused themes were examined through the concepts of acculturative stress, social support, psychological security, and social belonging.

NVivo 15 was used to organise interview and focus group transcripts and coded segments. The software helped manage coded data and compare themes across interviews and focus groups. However, interpretation was not produced by the software. The analytical interpretation was developed through close reading, memo writing, comparison across sources, and repeated movement between empirical materials and the theoretical framework (Table 2).

**Table 2:** Analytical procedure.

Step	Analytical action	Output
1	Repeated reading of interview and focus group transcripts	Initial notes
2	Open coding of legal uncertainty, language dependence, labour, ritual, support and belonging	Initial codes
3	Focused coding	Seven focused themes
4	Cross-source comparison	Interviews, focus groups and observation compared
5	Theoretical interpretation	Three-part framework: stress, support, psychological security
6	Negative case analysis	Ambivalent and contradictory cases retained

This analytical procedure helped connect empirical materials with the article’s main psychological concepts, especially acculturative stress, relational support, psychological security, and conditional belonging. For the present article, coding and theme development focused on how legal and administrative uncertainty, language dependence, gendered household expectations, and relational resources were linked to acculturative stress and psychological security. Belonging and local recognition were retained in the analysis when participants connected them to psychological security, social uncertainty, or gendered expectations.

### 3.7 Trustworthiness, reflexivity, and ethics

Several strategies were used to strengthen trustworthiness (Lincoln and Guba, 1985). First, methodological triangulation was used by comparing interviews, focus groups, and participant observation. Second, source triangulation was used by comparing accounts from wives, husbands, elders, family members, and other community members, while participant observation was used to contextualise these accounts. Third, contradictory cases were not removed. For example, some wives described support from family members, while others described pressure, control, or uncertainty within the same household setting. These differences helped refine the analysis and avoid presenting social support as always positive.

Follow-up clarification was used when possible. Key meanings were checked through further conversations with participants, while locally specific terms, ritual meanings, and ambiguous translations were clarified with trusted local contacts where needed. Reflexive attention was also given to how the researcher’s outsider position, language limitations, gendered interaction, and reliance on local contacts may have influenced access and interpretation.

Ethical issues were central to the study because some participants had sensitive legal or marital status. The study was approved by the Ethics Committee of the University of Malaya. Participants received information about the research aims, interview topics, voluntary participation, confidentiality, and their right to withdraw. Consent was obtained before interviews, focus group discussions, and audio or audiovisual recording.

Anonymity was protected throughout the study. Participants’ names were replaced with pseudonyms. Specific village names and household identifiers were omitted from the article. The article reports only broader township-level locations. The study did not collect identity card numbers, household registration numbers, border-crossing routes, or other highly identifiable legal information. Audio and audiovisual files and transcripts were stored securely. Only anonymised excerpts are used in the article.

## 4 Results: acculturative stress, relational support, and psychological security

This section presents three connected findings on acculturative stress, social support, psychological security, and social belonging among Akha-Hani cross-border wives. First, legal precarity, gendered household expectations, and language dependence produced culturally situated forms of acculturative stress. Second, family support, kinship networks, ritual guidance, and cross-border ties helped wives reduce uncertainty and build psychological security, while local recognition contributed to their developing sense of social belonging. Third, language learning, ritual participation, labour contribution, and intergenerational care became everyday routes to social belonging. These findings show that belonging was not gained through legal status alone. It was produced through repeated practices in households, villages, rituals, work, and cross-border family ties.

### 4.1 How legal precarity becomes acculturative stress

#### 4.1.1 Legal precarity and administrative dependence

Legal precarity was one of the clearest sources of acculturative stress. It did not appear only as an abstract legal condition. It entered daily life through registration, identity documents, parentage verification, police station visits, signatures, and access to public services. For cross-border wives and their families, documents shaped mobility, children’s schooling, health care, and long-term family planning. This made legal uncertainty a continuing psychological pressure.

This stress was most visible in documentation procedures. Ming Di from Daluo Township explained that DNA tests were required for household registration and access to public services. The cost of each test for parents and children was about 3,000 yuan. This created both economic and emotional pressure for cross-border families.

Ming Di’s account shows that legal precarity was not only about formal status. It also required families to prove marriage, parenthood, and family membership through repeated verification.

Language difficulties made documentation stress more concrete. Mandarin was needed for visiting hospitals, enrolling children in school, handling bank matters, signing papers, and communicating with officials. Yet language ability alone could not solve the problem when documents were incomplete. Da Pi described how his wife struggled to sign papers at the police station, so he had to complete parts of the process for her. In this case, administrative procedures increased her dependence on her husband.

Limited local recognition reduced some immediate pressure, but it did not remove legal precarity. Some cross-border women received Local Identity Recognition Cards from local police stations in Xishuangbanna. These cards helped with local mobility and daily affairs. A Ba described this support as useful because it allowed her to live more safely with her husband and reduced some uncertainty in village life. However, this form of recognition remained limited. It did not provide full legal identity or full access to public services.

Taken together, these cases show how legal precarity became psychologically consequential. Documentation problems did not generate acculturative stress simply because formal procedures were difficult. They reduced the predictability of residence, mobility, child registration, and access to services, while in some situations increasing reliance on husbands or other intermediaries. Legal precarity therefore entered everyday adaptation through uncertainty, dependence, and reduced control over routine family affairs. This helps explain why acculturative stress could persist even when women experienced considerable cultural familiarity within Akha-Hani households.

#### 4.1.2 Gendered expectations in the daughter-in-law role

Household adaptation was strongly gendered. The women in this study did not adapt as abstract migrants. They entered Chinese Akha-Hani households as wives and daughters-in-law. This position shaped what they had to learn, how they were evaluated, and where stress appeared in daily life. Adaptation involved domestic work, ritual participation, elder respect, agricultural labour, and the ability to act in ways that the husband’s family and village saw as proper.

For many women, this process began with festivals, family activities, and domestic rituals. These events were not only cultural occasions. They were also public moments in which a new wife could be observed by older women, in-laws, and neighbours. A Ba, a woman from Myanmar who married into Gelanghe Township, described this early experience:

When I first came to China, I found that Akha-Hani culture in Xishuangbanna was not the same as what I knew in Myanmar. This difference is most prominent in the festivals and family activities of the Akha-Hani ethnic group. For the Akha-Hani ethnic group in China, the festival had more steps and were more formal. As a woman, I had to participate in activities and do housework with older women. At first, I was not used to it and felt tired. But later I understood that these festivals are quite important for the village.

A Ba, 8 August 2024, Gelanghe Township, female.

A Ba’s account shows that shared ethnic background did not remove adaptation pressure. Although she was also Akha-Hani, village rituals in Xishuangbanna had different procedures and stronger expectations about women’s participation. Her stress came from unfamiliar ritual steps, gendered housework, and the need to act correctly in front of others.

Wedding rituals also worked as tests of family acceptance. A Ming explained that she had to take part in family sacrifices and follow several wedding procedures before entering her husband’s household. At first, she found these practices strange. Over time, she learned the local meaning of marriage through repeated participation and observation. Her experience shows that household adaptation was often learned through watching, correction, and practice.

Mistakes in ritual or domestic behaviour could carry social meaning. A wrong gesture, missed step, or uncertain response could be read as disrespect or unwillingness to join the husband’s family. This made ordinary etiquette stressful, especially when guidance was unclear. For cross-border wives, fear of making mistakes was tied to fear of not being accepted.

Family members often helped women learn these expectations. Older women showed them how to prepare food, receive guests, join rituals, and behave during festivals. Husbands explained some rules, but much learning took place through daily practice. This process was not quick. Women had to decide which local practices to follow, which earlier habits to maintain, and how to avoid conflict in the husband’s household.

These findings show that becoming a daughter-in-law formed an important part of acculturative adjustment. Women crossed a national border, but they also entered a gendered household order in which labour, ritual conduct, elder respect, and family reputation shaped everyday expectations. Shared Akha-Hani ethnicity reduced some cultural distance, yet differences in local procedures and daughter-in-law responsibilities could still produce fatigue, uncertainty, and fear of making mistakes. The psychological pressure therefore came less from complete cultural unfamiliarity than from learning how culturally familiar practices were organised and evaluated within a new household setting.

#### 4.1.3 Domain-specific language dependence and agency

Language operated differently across social domains. Shared Akha-Hani speech substantially reduced linguistic distance within many households and kin networks. Mandarin became more consequential when women moved beyond these settings into township markets, schools, healthcare services, banks, police stations, and other public or administrative contexts. Language dependence therefore did not arise from linguistic difference within the Akha-Hani community as such. It emerged when women moved between a culturally familiar kinship environment and settings that required different linguistic resources.

Da Pa from Gelanghe Township described this layered language situation when discussing his Burmese wife:

*My Burmese wife only knows Akha-Hani language and does not understand Mandarin very well. But Akha-Hani language is mutually understandable between China and Myanmar, and there are no communication barriers. Learning Mandarin is quite important for her to adapt to life in China. So I often persuade her to communicate more with the young people in the village (Da Pa, 20 July 2024, Gelanghe Township, male).*

Da Pa’s account shows that language had different meanings in different settings. Akha-Hani language made family communication possible and reduced isolation in the household. However, it was not enough for wider village and administrative life.

Language could also support belonging. In one focus group discussion, A Er explained that multilingual communication helped maintain harmony in cross-border marriage and strengthened cultural connection:

*Language is very important in our cross-border marriages. Many people here speak several languages. For example, I can speak Akha-Hani, Chinese, and a little Burmese. Like Ming Di here today, she speaks both Chinese and Akha-Hani, so when we eat, drink, and sing together, there are no communication barriers. She also sings many traditional songs in Akha-Hani for us (A Er, 3 July 2024, Daluo Township, male).*

This account shows that language was more than practical communication. It supported emotional inclusion. Singing, eating, drinking, and speaking across languages allowed women to join shared cultural moments rather than remain silent observers.

At the same time, limited Mandarin created dependence. Women often relied on husbands, relatives, or local helpers when they visited offices, hospitals, banks, or schools. This dependence did not always produce conflict, but it reduced their sense of independent action. It also reminded them that their safety in China was partly mediated through others.

The psychological meaning of language was therefore complex. Language learning increased confidence and reduced daily uncertainty. It also helped women enter non-kin relationships. However, language pressure could produce fear of mistakes, embarrassment, and silence in public settings. Participant accounts and contextual observation indicated that limited Mandarin could make some women hesitant to communicate beyond the village. Repeated everyday interactions with younger villagers and other local residents could gradually increase their confidence in public communication.

These findings indicate that language was not a uniform cultural barrier. Shared Akha-Hani supported family communication and cultural continuity, whereas Mandarin expanded women’s capacity to act independently in public and institutional settings. Limited Mandarin could intensify administrative dependence, but it was not itself the source of legal precarity. Its psychological significance lay mainly in agency. Women who could ask questions, explain their situation, and respond directly in public settings were less dependent on others and better able to manage everyday uncertainty.

### 4.2 Support mechanisms and psychological security

#### 4.2.1 Family and kin support as stress buffering

Family support was the first layer of social support. It came from husbands, in-laws, siblings, close relatives, and sometimes natal families across the border. This support helped women manage unfamiliar household routines and reduced some pressure created by legal uncertainty, language dependence, and village expectations.

Ming Da described family support as a source of inclusion. Her husband’s family in China often invited her to festivals and family meals. Her natal family in Myanmar also supported the marriage because they believed life in China offered more opportunities and convenience:

*I come from Myanmar, but my husband’s family in China is very welcoming. They often invite me to festivals and family meals. This makes me feel included and closer to them. My family in Myanmar also supports my marriage. They know life in China brings more chances and convenience (Ming Da, 20 August 2024, Damenglong Township, female).*

This account shows that support came from both sides of the border. For Ming Da, this double support reduced the emotional cost of relocation. It made marriage feel less like a complete break from her earlier life.

Practical support inside the husband’s family also reduced daily pressure. Da Ya explained that after her sister-in-law married into the family, she was treated as one of them. When Da Ya had time, she helped care for the children. This kind of care work was not only practical help. It also showed that the wife had been included in the family’s daily responsibilities.

Female peer networks were another important source of support. A Xi explained that building connections with other women who had married into the community helped her manage cultural unfamiliarity and homesickness:

*For me, the most effective coping mechanism has been building connections with other women who have also married into this community. We regularly gather to chat about our lives and sometimes share meals that combine both Myanmar and Xishuangbanna specialties. These gatherings give me a sense of belonging and help ease my homesickness (A Xi, 15 July 2024, Gelanghe Township, female).*

A Xi’s account shows that social support was not limited to husbands and in-laws. Women in similar marriages created spaces for emotional sharing, food exchange, and practical learning. These gatherings did not remove legal or household pressure, but they reduced loneliness.

Neighbourhood support also helped wives enter village life. Er Ni explained that neighbours helped his wife after she moved to the village. They taught her how to pick and process tea, and how to use an induction stove and gas for faster cooking because she had only known how to cook with a traditional fire pit before marriage.

Overall, family and kin support worked as a stress-buffering process. It helped wives handle unfamiliar routines, language barriers, childcare, and administrative dependence. It also gave them a social place from which they could act. Support was therefore not only something wives felt. It was also something done through meals, translation, childcare, work, and everyday companionship. Family and kin support worked as a stress-buffering mechanism because it made uncertain situations more manageable. However, this support also remained uneven because wives’ security often depended on husbands, in-laws, or village intermediaries. The protective value of support therefore depended partly on whether assistance expanded women’s ability to manage uncertainty or left them dependent on the same relationships for future situations.

#### 4.2.2 Ritual guidance and local recognition

Ritual guidance was another route to psychological security and village recognition. Festivals, wedding ceremonies, ritual meals, and domestic ritual spaces allowed cross-border wives to become visible in family and village life. These occasions also created moments when elders, in-laws, and neighbours could observe whether a wife understood local expectations.

Da You described how festival participation helped his wife develop a stronger sense of belonging. During important festivals, he and his wife went to the traditional headman’s house, pounded glutinous rice cakes, and joined the Swing Festival at the village gate:

*For example, during every important festival, we go together to the headman’s house. We pound glutinous rice cakes, and during the Swing Festival we go to the village gate to play on the swings. These experiences have helped her feel a stronger sense of belonging (Da You, 20 July 2024, Gelanghe Township, male).*

This account shows that belonging was produced through repeated participation. Through these practices, she became more visible and more included.

Ritual life was also connected with household authority and family memory. In focus group discussions, participants described family religious figures, Ghost Festival practices, and the fire pit as important parts of Akha-Hani domestic and ritual order. These details matter because ritual knowledge was tied to seniority, gender, and village authority. For wives from Myanmar and Laos, learning these rules was part of becoming accepted in the husband’s household.

Knowing where to sit, whom to greet, what food to prepare, and how to join festivals helped reduce social distance. These practices showed respect for elders and for the husband’s family. In this way, ritual participation became a public sign of commitment. It helped wives move from being newcomers to being recognised as family members.

At the same time, ritual recognition was not automatic. Elders and senior family members had more authority to judge whether a wife performed correctly. A wife could gain acceptance when she joined rituals and followed the expected order. However, the same rituals could also create pressure because mistakes were visible to others.

These findings reveal two related but analytically distinct processes. Ritual guidance functioned as informational and relational support because elders and senior family members helped women understand how to behave in socially visible situations. Local recognition referred to the social response that followed when a woman’s participation was acknowledged as appropriate, committed, or legitimate. Guidance could reduce uncertainty and fear of mistakes, whereas recognition could strengthen her social position in the household and village. At the same time, ritual participation remained demanding because performance was publicly evaluated. Ritual life could therefore provide support and recognition while also producing pressure.

#### 4.2.3 Cross-border ties and emotional continuity

Cross-border kinship ties provided emotional continuity after marriage. These ties did not simply pull women back to their places of origin. They helped reduce loneliness, maintain familiar cultural routines, and make adaptation in the husband’s village more sustainable. In this sense, support crossed the national border and remained part of daily family life.

Wu Er described cross-border family contact as ordinary border life. He explained that relatives from Myanmar, Laos, and Thailand often visited each other before the COVID-19 pandemic, and that these exchanges usually did not require formal documents. His account shows that cross-border kinship was locally understood as part of normal social life rather than as an exception. For wives, these ties reduced the feeling of being cut off from their original families.

Digital communication became especially important when physical visits became difficult. Mi Nei described how she stayed in touch with relatives and friends in Laos through WeChat groups. She also interacted with other Lao women who had married into China:

*When I first married into China, I enjoyed staying in touch with my relatives and friends in Laos, and we shared aspects of our daily lives in a WeChat group. I also liked interacting with other Lao women who, like me, had married into China. This helped me maintain a connection to Lao culture while adjusting to my new environment (Mi Nei, 10 August 2024, Gelanghe Township, female).*

Mi Nei’s account shows that digital contact was not only a technical tool. It supported emotional security and reduced isolation. By sharing ordinary moments with relatives and other Lao women in China, she maintained earlier cultural ties while continuing to adapt to her husband’s village.

Cross-border kinship support was sustained through small and repeated acts. These included visits, WeChat messages, shared photos, festival greetings, food exchange, and ordinary conversations. These acts helped wives remain connected to people who understood their earlier life. This support was quiet, but it mattered because it lowered anxiety and helped women continue adjusting to village life in China.

However, cross-border kinship did not replace local belonging. Wives still had to manage household expectations, participate in rituals, learn local speech, and respond to village evaluation. Cross-border ties and village recognition could therefore contribute to adaptation in different but complementary ways. Together, they show that social support in Akha-Hani cross-border marriages was built across households, villages, and digital communication. Cross-border ties helped wives keep emotional continuity with their earlier lives. This continuity reduced the sense of rupture after marriage and allowed local adaptation to develop without requiring a complete break from natal kin and previous cultural attachments.

### 4.3 Outcomes: psychological security and conditional belonging

#### 4.3.1 Psychological security through reduced uncertainty

Psychological security developed when wives felt less exposed to legal, social, and household risk. It was not a fixed internal state. It was built through practical help, trusted relationships, language learning, ritual guidance, and greater predictability in everyday interaction. For cross-border wives, feeling secure often meant knowing who could translate, who could explain official procedures, who could guide ritual behaviour, and who would protect them when mistakes occurred.

Legal uncertainty made this support especially important. Local Identity Recognition Cards, village mediation, police station registration, and family help did not fully solve legal precarity. However, they reduced some uncertainty in daily life. They helped wives move within local spaces, live with husbands, and manage basic affairs. In this sense, partial recognition had emotional effects even when it did not provide full legal status.

Psychological security also increased when wives gained more competence in local routines. Learning Mandarin, understanding festival procedures, managing household work, and interacting with neighbours helped women feel less dependent. These skills did not remove all stress, but they made daily life more predictable. They also helped wives act with more confidence in family and village settings.

Support from husbands and in-laws was especially important when legal and language barriers overlapped. When husbands translated official language, explained documents, accompanied wives to offices, or protected them in family disputes, wives could manage situations that might otherwise feel threatening. In these cases, social support worked by reducing exposure to risk.

However, the findings also show that psychological security could be fragile. If support depended too heavily on one person, such as a husband or elder, wives could remain vulnerable. If family relations became tense, the same dependence could become a source of insecurity. Participants’ accounts suggested that psychological security was more stable when women could draw on multiple sources of support rather than relying heavily on a single relationship.

These findings show that psychological security emerged from relational protection. It was not simply an individual feeling. It was produced when wives had people, practices, and local knowledge that helped them manage uncertainty. In the Akha-Hani borderland, security was built through everyday support rather than through legal status alone. Psychological security therefore emerged when uncertainty became shared and manageable. It was strongest when women could rely on several sources of support rather than on one person or one household relationship.

#### 4.3.2 Belonging through labour and family contribution

Labour contribution was one of the most visible ways through which cross-border wives entered household life. In villages where tea, rubber, fruit, and other agricultural work were central to family income, women’s labour was not treated as only economic. It also became a sign of commitment, reliability, and willingness to share family responsibility.

Liu Da described how his wife joined rubber and watermelon farming after marriage. Her labour reduced the workload for his parents and improved the family’s quality of life:

*After my wife married into my family, she joined me in rubber and watermelon farming, which has significantly reduced the workload for my parents and improved our overall quality of life (Liu Da, 20 July 2024, Gelanghe Township, male).*

Liu Da’s account shows that women’s labour helped them become recognised as useful members of the husband’s family. This kind of contribution helped create family inclusion.

Pi Er also emphasized labour sharing during the tea harvest. He explained that tea field work required both spouses, especially during the busy season. The work was physically demanding, but mutual support helped them manage it. His account shows that shared labour could create partnership and cooperation. It also shows that economic security was built through bodily effort and seasonal pressure.

Labour was linked with economic improvement. Some wives described better living conditions after marriage, including transport, housing, farming opportunities, and access to local services. Jia Hua said that after marriage her husband bought her a motorcycle, and the family later owned two motorcycles. She also described a plan to work hard and buy a family car within 5 years. For Jia Hua, transport was not only material improvement. It changed her mobility and gave the family a clearer sense of future planning.

At the same time, economic improvement brought continuing obligations. San Du explained in a focus group discussion that some women had heavier responsibilities to support families in Myanmar or Laos. Before 2022, sending money home was easier because the tea business in China was more profitable. In recent years, tea prices dropped sharply, making remittances a heavy burden for some families. This account prevents the analysis from presenting economic improvement as a simple success story.

These cases show that labour and economic participation had two psychological effects. Stable income, shared work, improved transport, and future planning could reduce uncertainty and strengthen women’s position within the household. At the same time, remittance pressure, market change, and continuing expectations from both marital and natal families could create new responsibilities and stress. Labour contribution therefore supported psychological security when it improved household predictability and cooperation, but it could become demanding when women’s value and future security were tied too closely to continuous economic contribution.

#### 4.3.3 Conditional belonging and gendered obligation

Belonging was produced through repeated participation, but it was not unconditional. Cross-border wives gained recognition by learning local language, joining rituals, contributing labour, caring for children, supporting elders, and taking part in long-term family projects. These practices helped them become accepted as wives, daughters-in-law, neighbours, and village members. However, recognition often depended on visible contribution and proper behaviour.

Intergenerational support made this conditional belonging especially clear. Older family members provided land, housing foundations, farming knowledge, childcare, and local guidance. Younger couples provided labour, income, elder care, and future planning. These exchanges were not formal contracts. They were rooted in family duty and village expectations.

Er Pa described intergenerational continuity through tea plantation work. He expressed his intention to return to his hometown and continue the family tea plantation business, so that the work and experience could be passed on to later generations. This account shows that household stability was not only about current income. It also depended on whether family land and farming knowledge could continue across generations.

Li Kai described a similar pattern in housing and rubber plantations. He explained that housing was allocated by the village committee, but younger family members had to work hard to build more modern and comfortable homes on existing foundations. He also noted that rubber plantations were inherited from parents.

For cross-border wives, these arrangements could increase belonging because they placed women within the long-term future of the husband’s family. A wife who contributed to farming, childcare, housing, and elder care was not only helping in the present. She was becoming part of the family’s future. This could strengthen her position and deepen social recognition.

However, the same process could also increase pressure. Once a wife became part of long-term household plans, she was expected to contribute not only to her husband, but also to elders, children, land, and family property. Her belonging was tied to responsibility. It was protective because it gave her a place in the family. It was also demanding because this place had to be maintained through continuing labour and moral conduct.

This tension is central to the findings. Social belonging helped wives reduce uncertainty and build psychological security, but it was shaped by gendered household expectations. In this sense, belonging was both a source of protection and a form of obligation. Conditional belonging therefore had a double psychological effect. It reduced uncertainty by giving women a recognised place in the household, but it also created pressure because this place had to be maintained through labour, care, ritual conduct, and public evaluation.

#### 4.3.4 Ambivalent and negative cases

Ambivalent cases also appeared in the data. Family support did not always remove insecurity. In some cases, help from husbands and in-laws reduced immediate pressure, but it also increased women’s dependence on family members in official procedures, public communication, and household decision-making. Village recognition also had clear limits. Being accepted as a wife or daughter-in-law could provide emotional protection, but this recognition often depended on visible labour, correct ritual behaviour, and respect for elders. When these expectations were difficult to meet, support could turn into pressure. Local recognition also did not replace formal legal status. Even when women were accepted by neighbours and relatives, uncertainty over documents, children’s registration, and access to public services continued to produce stress. These ambivalent cases show that social support and belonging should not be romanticised. They buffered acculturative stress, but they also reflected gendered obligation, household hierarchy, and the limited protection available under legal precarity.

These negative and ambivalent cases are important because they show the limits of social support as a protective factor. In this setting, support reduced stress only when it increased women’s room to act, rather than deepening dependence on family authority or uncertain local recognition.

These cases also clarify why family or village acceptance did not necessarily remove acculturative stress. Relational support and legal security addressed different sources of uncertainty. A woman could receive emotional protection and practical help from family members while remaining uncertain in state-facing procedures. Social support and local recognition therefore had genuine psychological value, but their protective effects had limits when legal uncertainty or dependence continued.

Overall, the results show that acculturative stress, social support, psychological security, and belonging were closely connected but analytically distinguishable. Legal precarity, gendered household expectations, and domain-specific language dependence created different forms of uncertainty and reduced agency. Family support, kinship networks, ritual guidance, peer relationships, and cross-border ties helped make these pressures more manageable. Labour, language competence, ritual participation, and intergenerational contribution could strengthen psychological security and social belonging, although their effects remained conditional when support depended on household authority, continuing performance, or unresolved legal uncertainty.

Table 3 brings together the key findings of the study. It shows that legal precarity, language dependence, ritual unfamiliarity, and labour expectations did not operate as separate stressors. They were connected with different forms of relational support, which helped reduce uncertainty and build psychological security. However, the table also shows that belonging was conditional because recognition depended on repeated participation in household, ritual, and village life.

**Table 3:** Stress conditions, relational resources, psychological processes, and psychological outcomes.

Stress condition	Relational resource	Psychological process	Psychological outcome
Uncertain registration	Husband translation, village mediation	Reduced immediate uncertainty but continuing dependence	Partial psychological security
Mandarin demands in public settings	Family members, peers, younger villagers	Greater communicative confidence	Increased situational agency
Ritual unfamiliarity	Elder or senior-family guidance	Reduced fear of mistakes	Greater predictability
Household role pressure	Shared work and family assistance	More manageable role demands	Reduced household strain
Cross-border separation	Natal kin, peer women, digital contact	Emotional continuity	Reduced loneliness

## 5 Discussion

This study examined how legal precarity, relational support, and local recognition shaped the psychological adaptation of Akha-Hani cross-border wives in Southwest China. Three findings are particularly important. First, substantial ethnic, linguistic, and ritual proximity did not eliminate acculturative stress when women continued to experience legal and administrative uncertainty. Second, social support reduced stress by making difficult situations more predictable and manageable, although support could also maintain dependence when women relied heavily on husbands, relatives, or local intermediaries. Third, local recognition could strengthen psychological security and belonging, but relational inclusion coexisted with continuing institutional vulnerability. Taken together, these findings show that acculturative adaptation in cross-border marriage is shaped by the interaction of cultural familiarity, institutional conditions, gendered relationships, and access to social support.

### 5.1 Legal precarity as culturally situated acculturative stress

Acculturation research has long identified cultural distance, language difficulty, identity negotiation, discrimination, and access to social resources as important conditions shaping psychological and sociocultural adaptation (Berry, 1997, 2001; Ward and Kennedy, 1999). More recent research has further linked acculturative stress with fear, psychological distress, emotion regulation, meaning-making, family relations, and wider settlement conditions (Bekteshi and Kang, 2020; Starck et al., 2020; López-de-León et al., 2025; Lerias et al., 2025; Soufi Amlashi and Forstmeier, 2025). The present findings support this contextual understanding of acculturative stress, but they also complicate an interpretation based primarily on cultural distance.

The Akha-Hani case is theoretically informative because many cross-border wives entered marital communities with substantial cultural familiarity. Shared Akha-Hani language, ancestry, kinship practices, and ritual traditions could reduce interpersonal distance and facilitate participation in household and village life. Yet cultural proximity did not produce equivalent security across all domains of adaptation. Women still encountered Chinese administrative procedures, Mandarin-dominant public settings, gendered household expectations, and uncertainty concerning documentation, mobility, and children’s future access to services. This pattern is consistent with research showing that marriage-migrant adaptation can develop differently across family, social, linguistic, and institutional domains rather than following a single linear trajectory (Yeung and Mu, 2020; Chen et al., 2021). Cultural proximity therefore reduced some adaptation demands without eliminating acculturative stress.

Research on China’s borderlands has documented unstable legal status and incomplete formal recognition among some foreign wives, while broader migration scholarship has shown how precarious migratory status can shape fear, dependence, mobility, and access to social rights (Barabantseva, 2018; Grillot, 2015; De Genova, 2002; Menjívar, 2006; Goldring et al., 2009). Regional research similarly shows that shared ethnic and cultural relations across Yunnan’s borders coexist with contemporary systems of registration and border governance (Shen, 2023; Xi, 2020). The present study adds a specifically psychological explanation. Legal precarity did not become acculturative stress simply because formal status was incomplete. It became psychologically consequential when uncertainty reduced the predictability of everyday life, increased reliance on husbands or other intermediaries, constrained women’s capacity to manage institutional encounters independently, and generated concern about residence, mobility, children, and future family security.

This distinction is theoretically important because legal precarity and acculturative stress operate at different analytical levels. Legal precarity refers to an institutional condition, whereas acculturative stress refers to the psychological strain that may emerge from living under that condition. The findings therefore identify uncertainty, dependence, reduced control, and constrained agency as mechanisms connecting institutional insecurity with psychological adaptation.

Language findings reinforce this domain-specific interpretation. Shared Akha-Hani speech often facilitated communication within households and kin networks, whereas Mandarin became more consequential in schools, healthcare settings, markets, administrative offices, and other public encounters. Limited Mandarin therefore did not necessarily indicate broad cultural distance. Rather, it could intensify institutional dependence when women needed assistance to understand procedures or communicate directly in state-facing settings. Language became psychologically significant through its relationship with agency: greater communicative competence increased women’s capacity to ask questions, explain their circumstances, and manage everyday institutional encounters with less reliance on others.

### 5.2 Social support as relational stress buffering

The second finding concerns the role of social support in managing acculturative stress. The stress-buffering model proposes that emotional, informational, and instrumental resources can reduce the psychological effects of stressful conditions (Cohen and Wills, 1985). Migration research similarly identifies family and social support as important resources in managing acculturative stress and facilitating adaptation (Bekteshi and Kang, 2020; Chen, 2025). The present findings extend this perspective by showing that support in rural Akha-Hani communities was enacted through culturally organised relationships and everyday practices rather than operating only as an individual perception.

Support was psychologically important because it changed the manageability of stressful situations. Translation helped women understand administrative and public communication. Practical assistance reduced demands associated with childcare, transport, agricultural work, and household responsibilities. Informational guidance made ritual procedures and local expectations more predictable, while emotional support from husbands, peers, and natal kin reduced isolation. These forms of support did not necessarily remove the original stressor. Rather, they provided resources for understanding and responding to it. In this sense, relational support strengthened psychological security by reducing uncertainty and increasing access to knowledge, assistance, and trusted relationships.

At the same time, the findings qualify an exclusively positive interpretation of social support. Assistance could reduce immediate stress while also maintaining dependence. When husbands translated official communication, accompanied women to institutions, or managed administrative procedures on their behalf, such assistance provided immediate protection. Yet repeated reliance on the same relationship could limit opportunities for women to communicate or act independently in later encounters. Support was therefore most protective when it increased women’s capacity to manage future situations, whereas support that permanently substituted for independent action could leave psychological security contingent on household relationships. This ambivalence is particularly important in gendered marriage migration, where care, protection, obligation, and dependence may coexist.

For analytical clarity, social support and local recognition should therefore be distinguished. Emotional, practical, and informational support refers to resources provided through relationships, whereas recognition refers to the social acknowledgement of a woman’s position as a legitimate family or community member. Recognition may strengthen psychological security, but it is not treated as a fourth category of social support.

### 5.3 Conditional belonging and gendered psychological security

The third finding concerns how local recognition and conditional belonging shaped psychological security. Social identity and group membership can provide social meaning, support, and a clearer sense of social position that contributes to coping and adjustment (Haslam et al., 2009; Hu and Cheung, 2024). In the present study, recognition as a wife, daughter-in-law, mother, neighbour, or participant in village life could reduce interpersonal uncertainty by giving women a more secure and socially acknowledged position within the husband’s family and community.

Conditional belonging does not imply that women’s attachments, family relationships, or experiences of acceptance were inauthentic. Rather, it refers to the social conditions through which membership was sustained and evaluated. Participation in household labour, childcare, ritual practice, kin obligations, language learning, and respect for senior family members often strengthened recognition. At the same time, these practices reflected gendered expectations concerning appropriate wifehood and daughter-in-law roles. Research on Asian marriage migration similarly shows that integration is shaped by gender systems and family relations that organise women’s positions across private, familial, and public spheres (Chang, 2020; Chiu and Yeoh, 2021).

Importantly, relational inclusion and structural vulnerability were not contradictory outcomes. A woman could be trusted by her husband’s family, participate confidently in local rituals, contribute to household life, and be recognised as a community member while still remaining insecure in state-facing procedures. Family and village recognition operated through kinship and local social relations, whereas documentation, public-service access, and formal residence depended on institutional recognition. This coexistence explains why psychological security could improve without legal precarity disappearing. Local recognition had genuine psychological value because it reduced interpersonal uncertainty, but it could not substitute for secure formal status.

Labour, caregiving, and ritual participation illustrate this dual process. These practices could strengthen recognition because they demonstrated involvement in household and village life, but they could also become sources of pressure when women’s social position was tied too closely to continued contribution or correct performance. Belonging was therefore psychologically protective without necessarily being institutionally complete or socially unconditional. It could strengthen confidence and reduce uncertainty while remaining embedded in gendered expectations and broader legal constraints.

### 5.4 Theoretical contributions to cultural psychology

Together, the findings make three contributions to cultural and migration psychology.

First, the study qualifies an interpretation of acculturative stress centred primarily on cultural distance. Shared Akha-Hani ethnicity, language, and ritual familiarity reduced some interpersonal and cultural barriers, yet administrative uncertainty, public language demands, and gendered household expectations continued to generate psychological strain. Cultural proximity should therefore be understood as one resource within acculturation rather than as a condition that necessarily removes acculturative stress.

Second, the study specifies how legal precarity becomes psychologically consequential. Institutional uncertainty affected adaptation when it reduced predictability, increased dependence on others, constrained independent action, and generated concern about family futures. This distinction separates the institutional condition of legal precarity from its psychological consequences and helps connect research on precarious migratory status with acculturative stress theory.

Third, the study develops a relational account of psychological security by distinguishing support from recognition. Emotional, practical, and informational support buffered stress by making difficult situations more manageable, whereas recognition strengthened security by giving women a socially acknowledged position within family and village life. Both processes could also have limits. Support could reproduce dependence, while recognition could remain tied to gendered expectations and could not substitute for formal legal security. Psychological adaptation was therefore relationally produced but structurally bounded.

These contributions also broaden the empirical settings through which cultural psychology examines adaptation. The Akha-Hani borderland illustrates forms of psychological adaptation that are closely organised through kinship, language, household responsibility, local recognition, and interaction with state institutions. This contextual perspective complements continuing efforts to broaden psychological research beyond populations and settings that have historically dominated the field (Henrich et al., 2010).

### 5.5 Practical implications

The findings have practical implications for reducing acculturative stress and strengthening psychological security among cross-border wives. First, administrative information should be made clearer and more accessible. Documentation, registration, parentage verification, residence recognition, and public service access can become daily sources of uncertainty. Clear explanations of procedures, especially in languages that women and families can understand, may reduce avoidable fear and dependence.

Second, support should not rely only on husbands and in-laws. Family support is important, but relying exclusively on family members may increase women’s dependence in public and institutional settings. Trusted translation assistance, village-level guidance, community mediation, and peer networks among women in similar marriages could help women manage official procedures, healthcare, school communication, and ritual participation with greater confidence.

Third, community recognition should complement rather than substitute for institutional security. Being accepted as a wife, daughter-in-law, mother, or village member can strengthen psychological security, but recognition may remain tied to expectations concerning labour, childcare, ritual participation, elder respect, and family responsibility. More stable documentation, clearer access to public services, multilingual administrative guidance, and community-based support could reduce the extent to which women’s security depends on continued performance within family relationships.

### 5.6 Limitations and future research

This study has several limitations. First, the data come from Akha-Hani communities in Xishuangbanna, especially Gelanghe, Damenglong, and Daluo. The findings cannot represent all cross-border marriages in China or Southeast Asia. Other border regions may have different legal arrangements, ethnic relations, labour patterns, and gender expectations.

Second, this qualitative study cannot estimate the prevalence of acculturative stress, psychological distress, or social belonging among all Akha-Hani cross-border wives. It also cannot establish statistical causal relationships between legal precarity, social support, psychological security, and belonging. Future research could combine ethnographic interviews with validated psychological scales to examine these relationships across a larger sample.

Third, the study focuses mainly on women who moved from Myanmar and Laos into Chinese Akha-Hani households. It does not provide a full comparison with women’s natal villages across the border. Future research could include fieldwork on both sides of the border to examine how household systems, legal expectations, and marriage practices differ across communities.

Fourth, language and translation may have shaped the data. Some interviews involved translation between Akha-Hani and Mandarin, and selected excerpts were later translated into English. Although meanings were checked against the field context and through follow-up clarification where possible, some emotional and culturally specific meanings may have been simplified. Future research could include more multilingual fieldwork and closer analysis of Akha-Hani terms related to marriage, stress, kinship, security, and belonging.

Fifth, purposive and snowball recruitment relied partly on trusted local contacts and participant referrals. Although several entry points were used and local contacts did not determine final inclusion, this recruitment strategy may have made socially connected households or participants more accessible than individuals with weaker village ties. Future research could diversify recruitment routes and include participants who are less connected to established family or community networks.

Overall, these limitations do not suggest that cross-border wives were unable to adapt. The findings instead point to a domain-specific process in which women could develop considerable competence in household, linguistic, economic, ritual, and social life while continuing to experience uncertainty in particular legal or institutional settings. Psychological adaptation should therefore be examined across relational, cultural, and institutional domains rather than treated as a single movement from non-adaptation to adaptation.

## 6 Conclusion

This article examined how legal precarity, relational support, and local recognition shape acculturative stress and psychological security among Akha-Hani cross-border wives in Southwest China. The findings show that substantial cultural proximity did not eliminate the demands of adaptation. Shared Akha-Hani language, kinship, and ritual traditions facilitated communication and participation in family and village life, while women continued to encounter uncertainty associated with administrative procedures, Mandarin-dominant public settings, gendered household expectations, mobility, and family futures.

Legal precarity became psychologically consequential when uncertainty reduced the predictability of everyday life, increased dependence on husbands or other intermediaries, constrained women’s capacity to manage institutional encounters independently, and generated concern about future family security. Social support helped buffer these pressures through emotional reassurance, practical assistance, information, translation, and culturally situated guidance. However, support was not uniformly protective. It could reduce immediate stress while maintaining dependence when women had limited opportunities to act independently.

Local recognition provided another source of psychological security. Recognition as a wife, daughter-in-law, mother, neighbour, or community participant could strengthen confidence and belonging by giving women a more secure social position. At the same time, this belonging remained conditional in the sense that it was often connected with gendered expectations concerning labour, childcare, ritual participation, elder respect, and family responsibility. Relational inclusion could therefore coexist with continuing institutional vulnerability, and community acceptance could not substitute for secure legal recognition.

The study contributes to cultural and migration psychology in three ways. It shows that acculturative stress can persist despite substantial cultural proximity, identifies uncertainty, dependence, reduced predictability, and constrained agency as processes through which legal precarity became psychologically consequential in participants’ accounts, and demonstrates that social support and local recognition can strengthen psychological security while remaining relationally and structurally bounded. The findings therefore support a multidimensional understanding of adaptation in which cultural familiarity, relational inclusion, institutional security, and individual agency may develop differently rather than as a single linear process.

## Data Availability

The datasets presented in this article are not readily available because the study involves human participants in a sensitive border context. Requests to access de-identified materials should be directed to s2109915@siswa.um.edu.my and will be considered in accordance with ethical and participant privacy restrictions.
